# Development of a Highly Permissive Mandarin Fish (*Siniperca chuatsi*) Kidney Cell Line for Mandarin Fish Ranavirus Using a Single-Cell Cloning Method

**DOI:** 10.3390/cells13010018

**Published:** 2023-12-20

**Authors:** Hetong Zhang, Junjian Dong, Yunyun Yan, Shanshan Liu, Xing Ye, Fengying Gao, Chengfei Sun

**Affiliations:** 1 Key Laboratory of Tropical and Subtropical Fisheries Resource Application and Cultivation, Ministry of Agriculture and Rural Affairs, Pearl River Fisheries Institute, Chinese Academy of Fishery Sciences, Guangzhou 510310, China; zhanght@prfri.ac.cn (H.Z.); dongjj@prfri.ac.cn (J.D.); 20222170546@pgs.hebau.edu.cn (Y.Y.); yexing@prfri.ac.cn (X.Y.); 2 Key Laboratory of Aquatic Animal Immune Technology of Guangdong Province, Pearl River Fisheries Institute, Chinese Academy of Fishery Sciences, Guangzhou 510310, China; 3 College of Oceanography, Agriculture University of Hebei, Qinhuangdao 066000, China; 4 School of Ecology, Sun Yat-sen University, Guangzhou 510275, China; liushansh@mail.sysu.edu.cn; 5 College of Fisheries and Life Science, Shanghai Ocean University, Shanghai 201308, China

**Keywords:** mandarin fish ranavirus (MRV), permissive cell line, single-cell clone

## Abstract

Mandarin fish ranavirus (MRV) infection poses a substantial challenge to the mandarin fish culture industry as no effective preventive or therapeutic measures currently exist. The creation of a highly permissive cell line from a natural host is crucial for developing a vaccine for MRV and understanding its pathogenic mechanisms. In this research, the mandarin fish (*Siniperca chuatsi*) kidney cell line (SCK) was isolated from mandarin fish kidneys. Subsequently, SCK-a to SCK-g monoclonal cell lines were derived from the SCK cell population, distinguished by morphological variations. Notably, MRV infection induced an advanced cytopathic effect (CPE) in almost all cells of the SCK-f clone. Further tests showed that MRV achieved a peak viral titer of 10^10.7^ 50% tissue culture infectious dose (TCID_50_)/mL and consistently exceeded 10^10^ TCID_50_/mL across nine passages in SCK-f cells. Electron microscopy verified the MRV virion integrity within SCK-f. In vivo experiments revealed that MRV infections led to cumulative mortality rates of 86.9% in mandarin fish and 88.9% in largemouth bass (*Micropterus salmoides*). Such results suggest that SCK-f is highly permissive to MRV. This study underscores the importance of cellular diversity in developing viral permissive cell lines. The SCK monoclonal cell line pool may offer potential for generating highly permissive cell lines for other mandarin fish viruses.

## 1. Introduction

Members of the *Iridoviridae* family contain a single linear double-stranded DNA genome, ranging from 99 kb (MG941005) to 212 kb (AF303741). Among the *Iridoviridae* family, the genera *Chloriridovirus*, *Iridovirus*, *Decapodiridovirus*, and *Daphniairidovirus* target invertebrates, while *Lymphocystivirus*, *Megalocytivirus*, and *Ranavirus* infect cold-blooded vertebrates. Lymphocystivirus infections result in low mortality in fish cultures. In contrast, typical infection by megalocytiviruses and ranaviruses lead to severe systemic diseases and mass mortality events.

Largemouth bass virus (LMBV), or Santee-Cooper ranavirus, was first identified as a causative agent in largemouth bass in the US during the 1990s and later spread across North America [1,2]. The pathogen has since expanded, both across host species and geographically. The review of the U.S. National Wild Fish Health Survey Data (2021–September 2023) revealed 30 fish species in which LMBV was detected. LMBV-like viruses have also been identified in Asia, including Thai ranavirus isolates in barcoo grunter (*Scortum barcoo*) and koi ranavirus in koi (*Cyprinus carpio* L.) [3,4], and LMBV’s presence in China was reported in 2011 under the name largemouth bass ulcerative syndrome virus [5].

The first known LMBV-like virus infection in mandarin fish occurred in 2017, with the isolated virus strain named mandarin ranavirus [6]. Mandarin fish ranavirus (MRV) shares a high genetic similarity to the LMBV North America isolate, with a 99.07% homology on major capsid (*MCP*) gene (MG941005.3, FR682503), and the two strains can be classified within the same genotype. The virus targets vital organs like the spleen, kidney, and intestines, and leads to significant fatalities, causing economic losses in mandarin fish cultures [7].

Notably, megalocytivirus-inactivated vaccines for red sea bream iridovirus (RSIV-I), infectious spleen and kidney necrosis virus (ISKNV-I), and RSIV-II (Aquavac IridoV, MSD) have been developed by using the highly permissive cell lines of mandarin fish fry-1 (MFF-1) or grunt fin (GF) [8,9], and subsequently received approval from the authorities in Japan, China, and Singapore, respectively. However, effective preventive measures against ranavirus infections like LMBV or MRV in largemouth bass and mandarin fish are yet to be established. Drawing from strategies against megalocytiviruses, an inactivated vaccine might provide a promising solution. For vaccine development and understanding pathogenic mechanisms, selecting suitable cellular substrates is crucial. Ideal cell lines for the study of ranaviruses should meet certain criteria: high permissiveness for virus replication to generate a substantial quantity of antigens capable of inducing an immune response; compatibility with continuous viral passages to establish virus banks for vaccine production and virology research; and derivation from the natural host to closely simulate the in vivo infection process. On the contrary, replication in cells with insufficient sensitivity would result in a low virus yield and may cause unexpected defects in the progeny virus, such as a loss of virulence [4]. However, only bluegill fry-2 (BF-2) cells have been demonstrated to be an LMBV titer exceeding 10^10^ 50% tissue culture infectious dose (TCID_50_)/mL among the tested fish cell cultures of fat-head minnow cells (FHMs), channel catfish ovary cells (CCOs), epithelioma *papillosum cyprini* cells (EPCs), chinook salmon embryo cells (CHSE-214), largemouth bass gonad cells (LMBGs), Chinese perch brain cells (CPBs), *Micropterus salmoides* heart cells (MSHs), and *Acanthopagrus latus* spleen tissue cells (ALS) [10,11,12,13,14]. This reflects the limitations of conventional strategies in developing highly permissive cell lines for ranaviruses, as these methods favor rapidly growing cells, leading to the gradual dilution of other cell populations in passaging. The reduced cellular diversity might cause unpredictability and challenges for establishing a highly permissive cell line for MRV.

In this study, we aim to establish a diverse monoclonal cell pool to develop a highly permissive cell line for MRV.

## 2. Materials and Methods

### 2.1. Development of SCK Cells 

A group of normal and apparently healthy mandarin fish with a body length of approximately 5 cm underwent sampling and PCR testing to exclude ISKNV or MRV infection. Subsequently, the selected donor fish was wiped with 70% alcohol for body surface disinfection. The kidney tissue, comprising only the mesonephros and metanephros, was surgically removed. It was then minced and suspended in 5 mL of 0.25% trypsin for 30 min. The resulting cell suspension was passed through an 80 μm mesh, centrifuged at 300× *g* for 3 min, and then resuspended in 5 mL of primary culture medium. This suspension was then inoculated into 25 cm^2^ cell culture flasks (Corning) and incubated at 27 °C in a 5% CO_2_ atmosphere. The primary culture medium contained 75% high-glucose Dulbecco’s Modified Eagle’s Medium (DMEM, Gibco, Waltham, MA, USA) and 25% Ham’s F12 (Gibco), and was supplemented with 20% fetal bovine serum (FBS, Gibco) and antibiotic-antimycotic 100× (Gibco) to a final concentration of 3%. 

Routine maintenance was conducted with high-glucose DMEM and 10% FBS. The cells reached confluence in 2–3 days, and then they were washed with Dulbecco’s Phosphate-Buffered Saline (Gibco) for a few seconds, followed by trypsinization with 0.05% trypsin–EDTA solution (Gibco) for 2 min. The detached cells were centrifuged at 300× *g* for 3 min at room temperature and then resuspended in the medium at a split ratio of 1:2. The suspension was then inoculated into cell culture flasks.

### 2.2. Generating Monoclonal Cell Lines 

SCK cells were trypsinized and disaggregated into individual cells by being passed through a serological pipe several times. Then, we quantitated the cell concentration with hemocytometers to prepare a dilute cell solution at a concentration of 10 cells/mL. The cell suspension (200 µL) was seeded into each well of a 48-well plate (Corning). After 12 h, wells with only one cell were identified and marked with microscopical analysis. The medium was refreshed every three days. After a week, wells showing single colonies were identified as monoclonal populations. The medium used for these single-cell clones was the antibiotic-free primary culture medium mentioned earlier. 

### 2.3. Virus Strain

The authors isolated the MRV strain from mandarin fish with symptoms, utilizing the SCK cell line. The identity of the MRV isolate was confirmed through sequencing of the viral genome. Cells and culture medium were harvested from MRV-infected SCK or SCK-f cells for use as the virus stock. Virus stock was stored at −80 °C.

### 2.4. Assessment of Cytopathic Effect 

SCK cells at the 40th passage were employed to assess the cytopathic effect (CPE) induced by MRV. Cells were passaged at an appropriate ratio, in triplicate, in 25 cm^2^ flasks to ensure a confluent monolayer at 48 h post passage, and then, were infected with MRV. The initial infectious dose was established at a multiplicity of infection (MOI) of 5. The virus culture was homogenized by three freeze–thaw cycles at 5 dpi. Subsequently, the homogenized virus culture was seeded into cells at a fixed ratio of 1:1000 (*v*/*v*) for serial passaging. Phase contrast images of SCK cells infected with the 3rd, 6th, and 9th passages of MRV were captured using a ZEISS Observer.Z1 inverted microscope at 3 dpi. Additionally, SCK clones, around the 15th passage, were inoculated with 6th-passage MRV at an MOI of 0.5, and images of the cells were captured at 5 dpi.

### 2.5. Virus Titer Assays 

To determine the virus titers of the 3rd, 6th, and 9th passages of MRV, SCK and SCK-f cells were seeded in 48-well plates with 200 μL of medium in each well. The MRV samples underwent 3 freeze–thaw cycles and were vortexed for 10 s for homogenization. Virus samples were then titrated using 10-fold serial dilutions, with six wells being inoculated with 200 μL of each dilution of each cell line. Wells were designated as positive if typical CPE was observed at 7 dpi. The TCID_50_ values were computed using the TCID_50_ calculator (Marco binder, University of Heidelberg, Heidelberg, Germany) based on the Spearman–Kärber method [15,16]. Virus titer values represent mean ± SE for three biological replicates of MRV samples.

To assay the titers of the 6th-passage MRV at various time points post infection, virus samples were collected at 1, 3, 5, and 10 dpi in an infectious dose with an MOI of 5, and the experimental methods are as described above. Virus titer values represent mean ± SE for 3 biological replicates of MRV sample.

### 2.6. Transmission Electron Microscopy

Cells were gently scraped from 25 cm^2^ flasks, centrifuged at 500× *g* for 3 min, and then fixed using 2.5% glutaraldehyde. Following osmium tetroxide post fixation, uranyl acetate was applied to enhance membrane contrast. Epoxy resin served as the embedding medium. The ultrathin sections were visualized using a Hitachi HT7800 transmission electron microscope [17]. 

### 2.7. MRV Challenge in Fish

Mandarin fish and largemouth bass, each weighing approximately 20 g, were sourced from local fish farms. Fishes were placed in isolation tanks for one week for quarantine and acclimation to laboratory breeding conditions, and they were randomly sampled for PCR testing of the viral *MCP* gene to confirm negativity for MRV. The primers designed for PCR testing were MRV-MCP-Q-1F: CACCACCTCTACTCTTA and MRV-MCP-Q-1R: ATGTTGTGGTTGATGGC. Then, groups of 15 fish were kept in 150 L tanks at temperatures ranging from 29 to 30 °C. Each fish received an intraperitoneal injection of 10^6.7^ TCID_50_ 6th-passage MRV suspended in 0.1 mL cell culture medium. Additionally, ten fish from each species were administered the same volume of untreated cell cultures to serve as controls. Fish mortality was monitored daily for 14 days, with cumulative mortality data collated from the three separate experiments.

### 2.8. Karyotype Analysis

Chromosome counts were carried out on 30th-passage SCK cells and 20th-passage SCK-f cells. Cells were grown in 25 cm^2^ flasks until they reached 70% confluence, then treated with colchicine at 3 μg/mL working concentration for 4 h at 27 °C. Then, cells were trypsinized and centrifuged at 300× *g* for 3 min. The cells were treated with 1 mL of 0.075 M KCL, tapped gently to disperse the cells, and then 0.075 M KCL was continuously added to reach a total of 4 mL and the mixtures were incubated at 27 °C for 20 min. Next, 6 mL of fresh methanol–acetic acid fixation was added for 5 min. The mixture was centrifuged at 300× *g* for 3 min, and 500 μL of the supernatant was left and tapped gently to resuspend, and the procedure was repeated twice. A total of 10 μL of cell suspension was dropped onto a glass slide. The chromosomes of at least 100 cells were counted under a Zeiss Axio Scope.A1 microscope.

### 2.9. Phylogenetic Analysis

The mitochondrial 16S rRNA gene and cytochrome oxidase subunit 1 (COI) genes were PCR-amplified from SCK cells and sequenced. The 50 μL PCR reaction mixture contained 25 μL of Q5 High-Fidelity 2X Master Mix (New England Biolabs, Ipswich, MA, USA), 2 μL each of 10 µM forward and reverse primers, and 50 ng of genomic template DNA. The thermal cycling program included initial denaturation at 98 °C for 30 s, followed by 30 cycles of denaturation at 98 °C for 10 s, annealing at 58 °C for 20 s, and extension at 72 °C for 45 s, followed by a final extension at 72 °C for 2 min. The primers used here were reported in previous studies and have been appropriately modified to match the *Sinipercidae* family:

mito16SUnivF-CGCCTGTTTACCAAAAACAT
mito16SUnivR-CCGGTCTGAACTCAGATCACGT [18,19]
mitoCOIUnivF-CGACCAATCACAAAGACATCGGCAC
mitoCOIUnivR-TAAGAAGCATTGTAATGCCAGCAGC [20].

The DNA sequence accession numbers used in phylogenetic analysis were: *Siniperca obscura* NC_021136.1; *Siniperca loona* KJ644781.1; *Siniperca fortis* NC_047290.1; *Siniperca roulei* NC_024432.1; *Siniperca scherzeri* JQ010987.1; *Siniperca undulata* KF815977.1; *Siniperca knerii* MK430069.1; *Siniperca chuatsi* NC_015822.1. Phylogenetic trees were constructed using PhyML with default parameters [21]. 

## 3. Results

### 3.1. Establishment and MRV Susceptibility of SCK Cell Line

**Figure 1:**
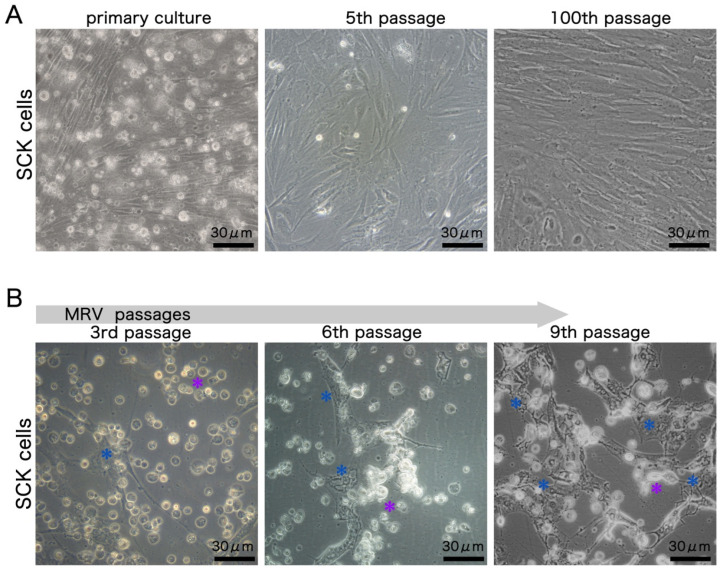
(**A**) Phase contrast microscopic views of SCK cells across different passages. The 5th passage shows diverse cell shapes without a prevalent type. By the 100th passage, fibroblast-like cells become predominant. (**B**) Phase contrast microscopic images detailing the CPE in SCK cells across virus passages at 3 dpi. Notable features of the typical MRV-induced CPE are cell shrinkage and rounding (denoted by purple asterisks), while the atypical CPE showcases varied shapes (denoted by blue asterisks). The share of atypical CPE progressively rises from the third to the ninth MRV generation.

The primary cells adhered well, displaying a spindle-like shape and a doubling time of roughly 48 h. By the fifth passage, cytomorphological analysis showcased distinct cell subtypes, including fibroblast-like, epithelial-like, and polygonal-like cells, pointing to a varied cell composition in the SCK cultures. However, by the 100th passage, fibroblast-like cells emerged as the dominant type (Figure 1A). 

Upon inoculation with the first-generation MRV, the advanced CPE appeared with 2 dpi, marked by cell shrinkage, rounding, and extracellular buildup of debris, culminating in cell lysis and detachment from the substrate. Clearly, the typical MRV-induced CPE was evident in almost all cells throughout the initial three infection cycles. Yet, as the virus underwent more passages, there was a discernible decrease in the potency of inducing typical CPE, reflected in the deferred onset of advanced CPE with 3 dpi in both the 6th and 9th passages and a gradual rise in the number of atypical CPE cells. Atypical CPE characteristics were vacuoles within cells, the emergence of filopodia-like structures, and sporadic inclusion bodies, hinting that the SCK cells might exhibit limited permissiveness to MRV (Figure 1B).

### 3.2. Development of Monoclonal Cell Lines

**Figure 2:**
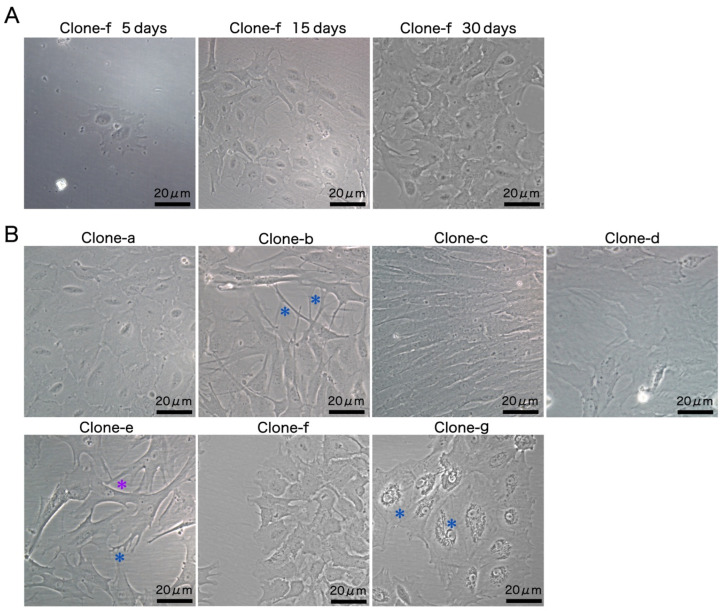
(**A**) The growth of clone-f. Initial cell cloning revealed slow growth, followed by an accelerated proliferation rate, resulting in monolayer formation within roughly 30 days in a single well of a 24-well plate. (**B**) The phase contrast images depict seven cell clones derived from the SCK cell line, each with unique morphological characteristics: clone a: an epithelial-like morphology; clone b: a multipolar shape with dendritic structures (blue asterisks); clone c: fibroblastic-like morphology; clone d: flat and irregular shape; clone e: multipolar morphology with cytoplasmic processes (purple asterisk) and dendrites (blue asterisk); clone f: polygonal shape approximately 20 μm in diameter; clone g: polygonal morphology with perinuclear vacuolation (blue asterisks).

With the primary objective of cultivating a highly permissive cell line for MRV propagation, monoclonal cells with various cellular morphologies were derived from SCK cells. The growth of clone f (SCK-f) was monitored to delineate the development of monoclonal cell lines. During the initial stages of culture, SCK-f exhibited sluggish growth, taking roughly 15 days to proliferate into a compact cluster comprising tens of cells, while following the first passage, there was a noticeable acceleration in the growth rate and then the cells culminated in a monolayer formation at around 30 days (Figure 2A). 

Each of the seven monoclonal cell lines displayed distinctive characteristics during the logarithmic growth phase. Clone a exhibited a thin, flat, plate-like morphology, approximately 25 μm in diameter, suggesting an epithelial origin. Clone b had a multipolar shape with dendritic structures. Clone c displayed an elongated spindle shape, characteristic of fibroblasts. Meanwhile, clone d manifested as large, flat, and irregularly shaped cells, with less conspicuous nuclei and discernible fibers within the cells. Clone e presented a multipolar form with cytoplasmic processes and dendrites, accompanied by a nucleus with high light transmittance and defined edges. The distinctive features of the clone f monoclonal cell line included cytoplasmic processes and a large cell nucleus, with diameters of around 20 μm. Lastly, clone g showcased a polygonal morphology with perinuclear vacuolation (Figure 2B).

### 3.3. MRV Sensitivity of Monoclonal Clones

**Figure 3:**
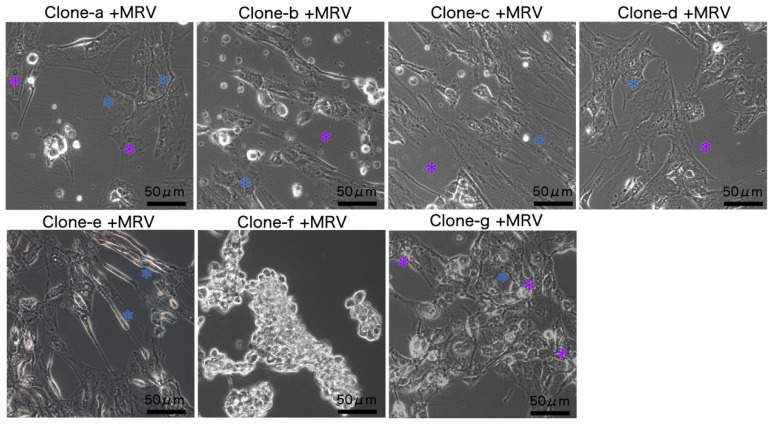
The MRV-induced CPE in various clones at 5 dpi. In clone a, there are noticeable cytoplasmic radial fibers (blue asterisks) and pyknotic nuclei (purple asterisks). Clones b, c, and d have evident inclusion bodies (blue asterisks) along with extracellular debris (purple asterisks). Clone e displays elongated swelling (blue asterisks), while clone f presents the typical CPE. Lastly, clone g showcases vacuoles (purple asterisks) coupled with filopodia-like structures (blue asterisks).

The CPE in monoclonal cells was studied to determine how MRV infection varied between them. Using a multi-step replication strategy with an MOI of 0.5, the clones were exposed to MRV. Observations at 5 dpi revealed that only clone f exhibited a typical MRV-induced advanced CPE in almost all cells, suggesting high permissiveness to MRV. In contrast, the other six clones (clones a to e, and g) predominantly showed atypical CPE. Cells exhibiting atypical CPE often had pleomorphic morphologies; apart from this, variations among the clones included radial fibers in the cytoplasm and pyknotic nuclei in clone a; evident inclusion bodies and extracellular debris in clones b, c, and d; elongated swollen cells in clone e; and vacuoles with filopodia-like structures in clone g (Figure 3).

### 3.4. Characteristics of MRV Infection in SCK-f and In Vivo

**Figure 4:**
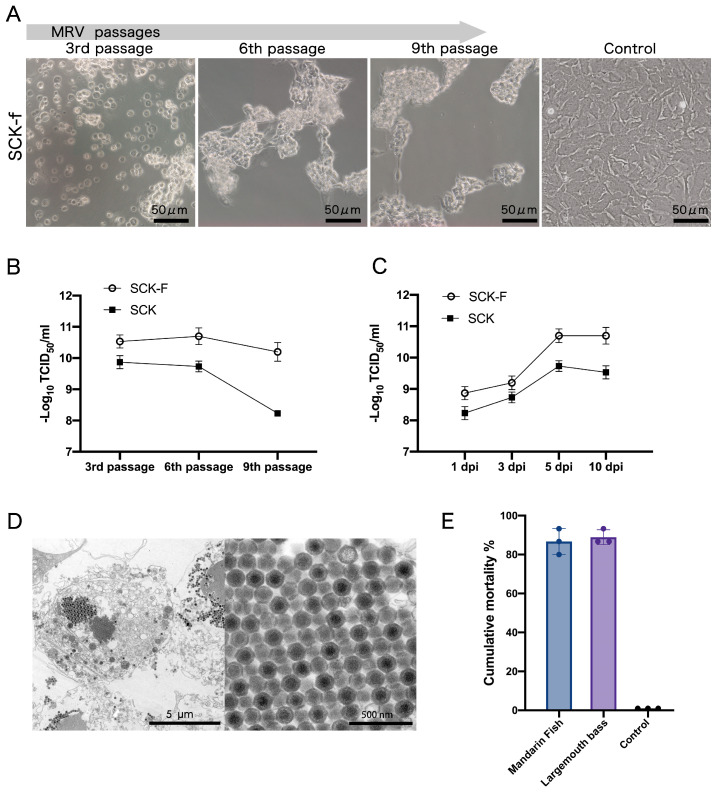
(**A**) Phase contrast microscopic images depict CPE in SCK-f cells elicited by the 3rd, 6th, and 9th passages of MRV at 3 dpi. (**B**) Comparative infectious dynamics of MRV in SCK and SCK-f cells. Viral titers of the 3rd, 6th, and 9th passages of MRV are expressed as TCID_50_/mL. Each titer value represents the mean ± SE derived from three biological replicates. (**C**) Comparison of the MRV replication dynamics in SCK and SCK-f. Growth curves were generated by measuring the viral titers at 1, 3, 5, and 10 dpi and were expressed as TCID_50_/mL. Titer values represent mean ± SE for 3 biological replicates. (**D**) Transmission electron micrographs of the MRV-infected SCK-f cells, revealing the substantial number of virions characterized by a hexagonal cross-section and an electron-dense core. Some of these virions demonstrate a crystalline array. (**E**) The cumulative mortality rates in MRV-infected mandarin fish and largemouth bass; mean ± SD from three independent experiments.

MRV replication was investigated in the SCK-f cell line by measuring viral titers, assessing CPE, and monitoring stability over multiple passages. MRV was serially passaged in the SCK-f cell line, and consistently inducing the typical CPE from the first to ninth passages around 2 dpi. They were markedly distinct from the atypical CPE observed within SCK cells (Figure 1B and Figure 4A).

We also compared MRV titers in SCK and SCK-f cells. In SCK, the titers at passages three, six, and nine were 10^9.87^, 10^9.73^, and 10^8.23^ TCID_50_/mL, respectively, marking an almost 45-fold decrease from the third to the ninth passage. Meanwhile, those in SCK-f cells were 10^10.53^, 10^10.7^, and 10^10.2^ TCID_50_/mL. In comparison, SCK-f had considerably higher titers than SCK at each passage with a 4.6-, 9.3-, and 93.3-fold increase (Figure 4B).

Viral growth curves within the SCK and SCK-f cell lines were then established by assessing the sixth-passage virus titers at 1, 3, 5, and 10 dpi. Peak titers were noted at 5 dpi in both cell lines. Specifically, the replication rates in SCK-f were higher than those in SCK at all four time points, increasing by approximately 3–9.3 times (Figure 4C).

Using transmission electron microscopy, we studied the ultrastructure of MRV in SCK-f cells. The images revealed that the virions had a hexagonal profile and a dense core with a crystalline array, while no structurally defective virion was found (Figure 4D). The virulence of the sixth-passage MRV from SCK-f was subsequently assessed in fish. Both mandarin fish and largemouth bass exhibited a series of characteristic symptoms following MRV infection, including anorexia, progressively worsening balance disorder, and the accumulation of ascites. The mortality rates reached 86.9% and 88.9% within one week. The subsequent absolute quantitative PCR testing confirmed the MRV infection. In contrast, the control group showed no deaths over a 14-day observation period. These results suggest that MRV propagated in SCK-f displayed a high virulence (Figure 4E).

### 3.5. Karyotype and Phylogenetic Analysis

**Figure 5:**
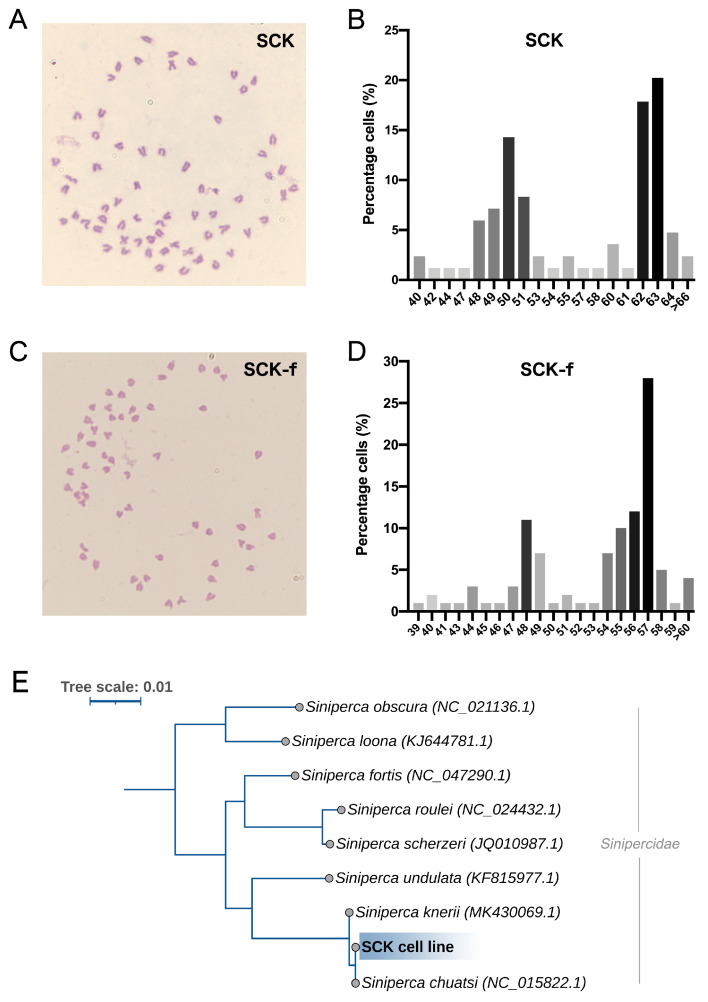
Giemsa staining was used to label chromosomes in SCK and SCK-f cells, with statistical analysis conducted on 100 cells per group. (**A**) Image illustrating SCK cells containing 63 chromosomes. (**B**) Chromosome number distribution in SCK cells. (**C**) Image illustrating SCK-f cells with 57 chromosomes. (**D**) Chromosome number distribution in SCK-f cells. (**E**) Phylogenetic tree of mitochondrial 16S and COI genes from SCK and eight *Sinipercidae* species.

The 30th-passage SCK cells had chromosome counts ranging from 40 to 106, with a trimodal distribution of modal numbers at 50, 62, and 63, together making up 52.4% of the population (Figure 5A,B). On the other hand, the 20th-passage SCK-f cells had chromosome counts between 39 and 111, with a modal number of 57, comprising 28% (Figure 5C,D). Serving as the baseline, the diploid chromosome number of the mandarin fish is 2n = 48. The variance in modal numbers between the two cell lines implies that SCK-f is not the prevalent population in SCK. Notably, even with the different karyotypes in SCK-f cells, the advanced CPE remained consistent across all cells. To further understand the role of chromosome ploidy on MRV susceptibility in SCK-f, we conducted another round of single-cell cloning from SCK-f. Twenty-four randomly selected clones, speculated to have different chromosomal modes, underwent a multi-step replication analysis. There was no difference in the timing of the advanced CPE outbreak across these clones, and all cells showed typical CPE, suggesting no link between chromosome ploidy and susceptibility. To gauge the genetic divergence from the reference strain of the *Sinipercidae* family, we amplified the SCK cell mitochondrial *16S* and *COI* genes. Phylogenetic analysis showed alignment with the *Siniperca chuatsi* species, with an entirely identical nucleic acid sequence (Figure 5E).

## 4. Discussion

Studies on MRV in vivo infection have revealed that kidney tissue produces a high viral copy number [7]. This observation led us to develop the kidney-derived SCK cell line. Yet, when assessing its susceptibility to MRV, a declining viral titer was noted over increasing passages, with only a subset of cells displaying typical CPE. The current research underscores the complexity of fish kidney cellular composition. For instance, in zebrafish kidneys, at least seven unique clusters have been discerned through single-cell RNA sequencing, which includes three epithelial cell types from the proximal, intermediate, and distal tubules, as well as mucinous cells, kidney stem/progenitor cells, multiciliated cells, and vascular endothelium cells [22]. Interestingly, even during fatal infections, MRV remains restricted to certain cells within target organs, notably the renal tubules [7]. We proposed that, similar to the kidney, only a specific group of cells within the diverse SCK cell population is susceptible to MRV. Therefore, seven monoclonal cell lines with distinct morphologies were derived from SCK cells, collectively forming a monoclonal cell pool. The method effectively countered the predominance of fibroblast-like cells in SCK cultures over successive passages, thereby preserving cellular diversity reminiscent of tissue composition. Both SCK and SCK-f cells have undergone over 100 generations, suggesting they may be immortalized through spontaneous transformation.

The prior study suggests that only cells manifesting the typical CPE are permissible for RSIV [23]. Following this premise, SCK-f, which demonstrated the typical CPE exhibited in almost all cells, was chosen for thorough testing. As anticipated, SCK-f proved to be highly permissive to MRV, given its peak titer of 10^10.7^ TCID_50_/mL. By comparison, BF-2 cells, recommended for LMBV isolation by the American Fisheries Society Fish Health Section, yielded a viral titer of 10^10.5^ TCID_50_/mL [10]. The Chinese perch brain cell line (CPB) registered a titer between 10^7.72^ and 10^8.13^ TCID_50_/mL for Santee-Cooper ranaviruses, and the largemouth bass ovary cell line logged 10^8.47^ TCID_50_/mL [11,12]. While the MFF-1 and ALS cell lines (sourced from yellowfin seabream spleen tissue) have been utilized for MRV cultivation, their responsiveness to MRV or LMBV remains uncharacterized [7,14].

Analyzing consecutive infection titers has been adopted as an indicator of megalocytivirus permissibility in cells [24]. Cultivating RSIV in non-highly permissive cells like BF-2 can cause rapid titer drops, such as in [25]. Another detailed study noted that cultivating the grouper iridovirus of Taiwan (TGIV) in KRE cells led to a titer decline from 10^6^ to 10^1.9^ TCID_50_/_mL_ within just five passages [26]. Thus, we employed this method to gauge the MRV permissibility of SCK-f. Significantly, SCK-f not only delivered enhanced MRV titers but also maintained titer stability over extended passaging. After validating the MRV virion integrity in SCK-f via electron microscopy, we further confirmed virulence in mandarin fish. The observed mortality rates closely matched the average value of 79.3% reported from five independent outbreaks in aquaculture farms [7]. 

The lack of discernible biomarkers makes it challenging to distinguish clones that share a similar morphology, while such clones may have varied transcriptional profiling. Discarding them would result in diminished cellular diversity. Hence, solely depending on cell morphology to develop specific-pathogen-permissive cell lines has its limitations. Indeed, even cells with identical morphologies can vary in their susceptibility to viruses. For instance, both the fibroblast-like cell lines from grunt fin (GF) and the red sea bream tail fin (CRF-1) are susceptible to RSIV, while another two fibroblast-like lines from the same species and tissues resisted the infection [25]. Another observation was the derivation of six single cell clones from SSN-1; clones with the same morphology exhibited different susceptibilities to four nervous necrosis virus (NNV) strains [27]. It might be prudent to create a non-biased monoclonal cell pool (i.e., randomly selecting the clones) to identify highly permissive cells. However, this strategy poses the challenge of managing and maintaining tens or hundreds of single-cell clones. Constructing a monoclonal cell pool based on cellular morphology offers a streamlined alternative, balancing diversity with convenience. Thus, such a pool is easy to maintain or cryopreserve, as this provides advantages for other research purposes. Studies have indicated that the kidney is the target organ of multiple mandarin viruses, such as infectious spleen and kidney necrosis virus and rhabdovirus [28,29]. Based on this fact, we inferred that a kidney-derived monoclonal cell pool may hold the potential for developing other virus-sensitive cell lines. Our next step involves examining other mandarin fish viruses to validate the universality of this strategy.

In summary, the approach in our study provides insights into the establishment of diverse clone cell lines from single tissues and the selection of a highly permissive SCK-f cell line for MRV replication, contributing to the development of other highly permissive cell lines and laying the groundwork for future MRV research endeavors.

## Data Availability

The cell lines metadata presented in this study are openly available in the NCBI BioSample database. SCK cell line: [Accession Number: SAMN38845202] (Link: https://www.ncbi.nlm.nih.gov/biosample/38845202); SCK-f cell line: [Accession Number: SAMN38845369] (Link: https://www.ncbi.nlm.nih.gov/biosample/38845369).
